# Jumping on the Bandwagon: A Review on the Versatile Applications of Gold Nanostructures in Prostate Cancer

**DOI:** 10.3390/ijms20040970

**Published:** 2019-02-23

**Authors:** Monira Sarkis, Esther Ghanem, Kamil Rahme

**Affiliations:** Department of Sciences, Faculty of Natural and Applied Sciences, Notre Dame University-Louaize, ZoukMosbeh P.O.Box:72, Lebanon; mmsarkis@ndu.edu.lb (M.S.); eghanem@ndu.edu.lb (E.G.)

**Keywords:** gold nanoparticles, prostate cancer, theranostics, in vitro and in vivo studies, nanotherapy

## Abstract

Prostate cancer (PCa) has remarkably emerged as a prominent disease in the face of the male population. Conventional treatments like prostatectomy or radiation can be curative only if PCa is diagnosed at an early stage. In the field of targeted therapy, a bevy of novel therapeutic approaches have left a landmark in PCa treatment and have proven to extend survival via distinct modes of actions. Nanotherapy has started to take root and has become the hype of the century by virtue of its abundant advantages. Scientists have invested a great deal of interest in the development of nanostructures such as gold nanoparticles (AuNPs), which hold particularly great hope for PCa theranostics. In this article, we present an overview of the studies published after 1998 that involve the use of different functionalized AuNPs to treat and diagnose PCa. Special reference is given to various in vitro and in vivo methods employed to shuttle AuNPs to PCa cells. Major studies show an enhancement of either detection or treatment of PCa when compared to their non-targeted counterparts, especially when AuNPs are tagged with specific ligands, such as antibodies, tea natural extracts, folate, anisamide, receptor inhibitors, and chitosan. Future approaches of treatment are dependent on those worthy multifunctional molecules, and are dictated by their ability to achieve a more versatile cancer therapeutic approach.

## 1. Introduction

**Figure 1:**
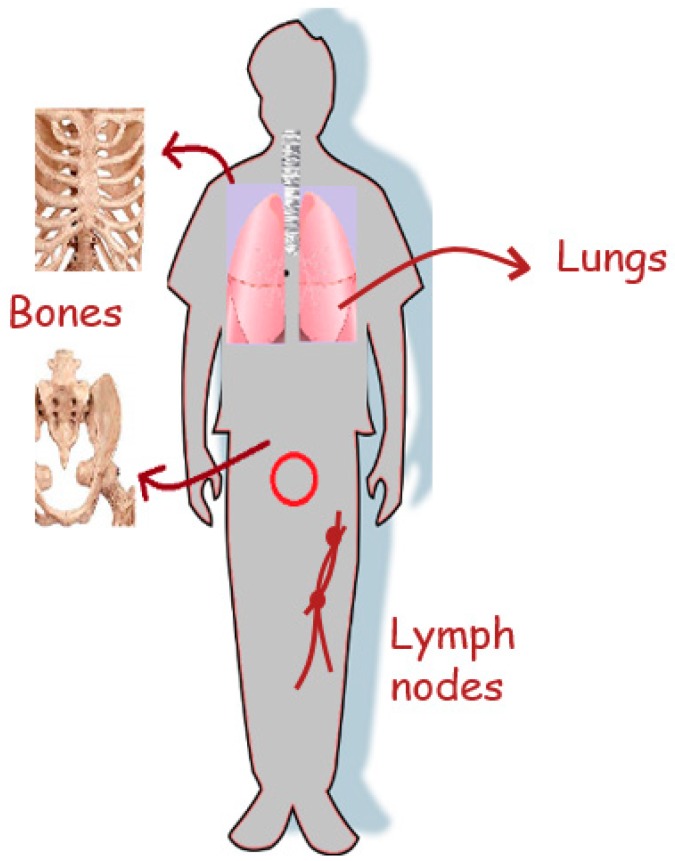
Representative image of the primary locations of prostate cancer (PCa) metastasis.

Prostate cancer (PCa) occurs in the prostate gland that adheres firmly to the base of the bladder and has a fundamental role in producing and secreting the prostate fluid that mainly functions to cushion and nourish the sperm with the seminal fluid, along with other constituents such as enzymes and lipids [1]. PCa affects the prostate gland upon the over-expression of certain receptor molecules, such as prostate specific membrane antigen (PSMA) and laminin. Several predisposed factors such as age, family history, and obesity might serve as stimulants. It has become by far the most common malignancy in the face of the male population, for it is the second most frequently diagnosed cancer in men worldwide [2]. Despite the fact that PCa is a slow-growing cancer, it can become aggressive when metastasis occurs and spreads throughout the body. PCa metastasis mainly occurs to the bone, lymph nodes, and lungs [3], as depicted in Figure 1. The treatment of PCa has been a subject of controversy for years. Typically, surgery and radiation therapy are two established protocols that are done when the cancer is local and confined to the prostate gland [4]. Radical prostatectomy, hormone therapy, chemotherapy, radiotherapy, and combination therapy are ordinarily options referred to when the disease is advanced (aggressive) and has spread to nearby tissues. Some of which include either the removal of the prostate gland, treating with one or two different chemotherapy drugs to enhance the outcome, or applying radiation such as x-rays to defeat the tumor. Treatments at this time frame are only embarked to palliate the symptoms of the disease [5] while masking the health side effects left post-treatment. For instance, the major demeriting side effect of chemotherapy lies in the non-selective uptake of drugs by healthy cells [6]. Moreover, having a complete successful toxicity on PCa through targeted radioimmunotherapy (RIT) remains out of reach [7,8]. There is a need for the development of existing therapies while prioritizing and placing targeted therapy as a top-notch asset in improving the efficacy of treatments and increasing the survival rate of patients [9]. In the next sections, we briefly visit the world of nanotechnology as a transit to the arena of gold nanoparticles (AuNPs) and their crucial role in PCa theranostics [10].

## 2. Nanotechnology and Its Importance in Theranostics

The term “nanotechnology” is often used to define studies that deal with substances that have very small dimensions, which are usually in the nanometer range, and such particles are coined with the term “nanoparticles”. Particles at a nanometer scale are well known to exploit different properties from bulk materials, which arise from the interplay of classical physics and quantum mechanics [11]. Nanomaterials have underlying principal parameters that give them unique physiochemical properties which are determined by their size, shape, and composition. Such parameters are projected to have a wide range of applications, from targeted drug delivery to biomedical imaging, and more recently to personalized medicine [12].

The use of nanoparticles that are generally less than 100 nm in size has increased exponentially in recent years [9,13]. The key benefits of nanotherapy can be disentangled into two major objectives, namely, maximized drug loading capacity due to their high surface-area-to-volume ratio, and increased tumor uptake through prolonged drug circulation and lessened risk of undesirable toxic effects to nearby healthy tissues [14,15]. Moreover, to overcome the mutability of cancer, it is critical to achieve an idealistic drug delivery vehicle that is characterized by long blood circulation time, and specific targeted therapy consists of clinically approved components to effectively combat the tumor within a short time frame [16]. This notion can be exemplified and put into perspective by the ample studies done on using nanomaterials in theranostics for different types of cancers [17,18]. Thus, nanotechnology has rapidly emerged in the field of medical imaging and targeted drug delivery [19]. The uses of nanoparticles, for example as both imaging and targeting agents, may be coalesced to form a single entity capable of performing dual functions, and therefore with the use of nanotechnology, the pitfalls in the current approaches for treatment would be eliminated and rectified. This groundbreaking discovery and multifaceted world of nanosystems will be set on a pedestal and will prove to be very promising for various medical applications, for it has already started to influence the way diseases, specifically cancer, are addressed. Overall, the benefit to patients undergoing chemotherapy is sufficiently low and is outweighed by the risks and harm stemming from treatment, tipping the balance in favor of targeted alternative treatments and accentuating their importance [20]. The major advantage displayed by targeted therapy is the delivery of drugs specifically to cancer tissues. This feature allows it to be at the forefront relative to conventional chemotherapy regimens [21,22]. In targeted therapy, having a uniquely over-expressed antigen on the tumor of interest is of paramount importance, since it reinforces the targeted delivery of the drugs and spares normal tissue.

**Figure 2:**
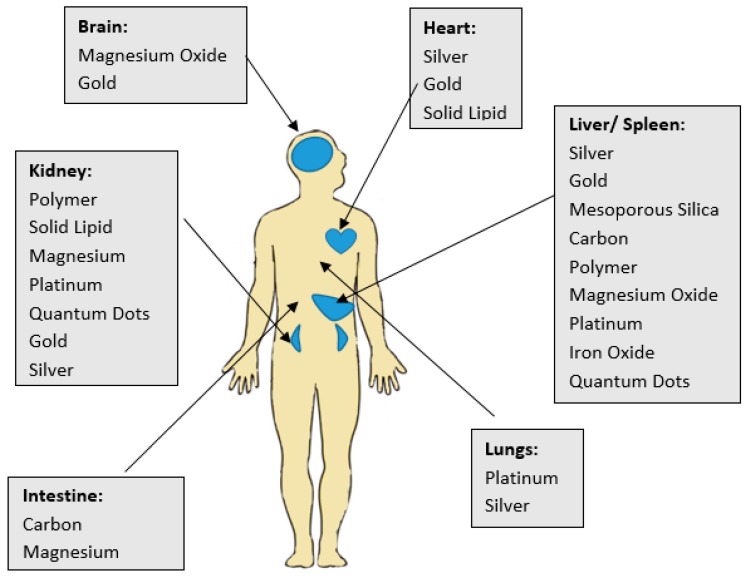
Summary of the distribution of nanoparticles (NPs) in vivo.

Throughout the years, different types of nanosystems have been addressed in order to study their various biological applications and have a better understanding on which particles serve the human kind best in terms of serious diseases such as cancer [15]. Regarding the enhancement of PCa diagnosis and treatment, among the different types of nanoparticles studied so far are iron oxide, silver, gold, platinum, quantum dots, and liposomes [23,24,25]. The main challenges in the methods using the above-mentioned nanoparticles are to overcome their limited biodistribution, toxicity, and clearance from the excretory pathway [13] that is depicted in Table 1. A summary of the distribution of nanoparticles (NPs) in vivo is represented in Figure 2.

Narrowing down on the vast and innumerable therapeutic drug delivery vehicles that have been employed, AuNPs have been greatly explored due to their enhanced biodistribution and their capacity to accommodate large payloads [26]. The use of AuNPs holds a crucial impact in the field of nanotechnology and in shaping the future of targeted cancer therapy, as discussed below.

## 3. Attractive Properties of Gold Nanoparticles

Researchers have made quite an improvement in finding solutions to overcome the obstacles facing current treatment approaches. However, we are still far from “hitting the nail on the head” in terms of truly finding an efficient method for drug delivery and without serious side effects. One essential factor that one ought to bear in mind is that the complexity of the synthesis of any nanoparticle plays a crucial role in determining its efficient use and practicality. Many nanoparticles are difficult to produce in aqueous media, such as quantum dots, metallic nanoparticles, ceramic nanoparticles, oxide nanoparticles, and many more (by having multiple processes that should be undergone and difficulty controlling the reactions, hence consistency in the product is not always achieved). On the contrary, AuNP synthesis in water is “praised” for its ease of production, with a high control of the size and shape-dependent optical properties, which in turn allows for a wide biofunctionalization of the AuNPs due to the high affinity for thiols and amino groups present in biomolecules. Their ability to be manipulated—also provides a versatile platform for nano-biological assemblies with oligonucleotides, antibodies, and proteins, with their high surface area allowing for a dense presentation of multifunctional moieties serving as practical platforms for applications in biology and medicine [27,28]. Furthermore, AuNPs are also known for having no or very low cytotoxicity when compared to other types of nanostructures (i.e., quantum dots) [29]. AuNPs have proved to be a multifunctional platform because they can be used for bioimaging and therapeutic functions [27,30]. This is mainly due to their inherent capability to exert optimal therapeutic effects with minimum leakage of payload away from target sites, and also because their nano-dimensions can be easily controlled and designed to match the sizes of tumor vasculature [31,32].

**Figure 3:**
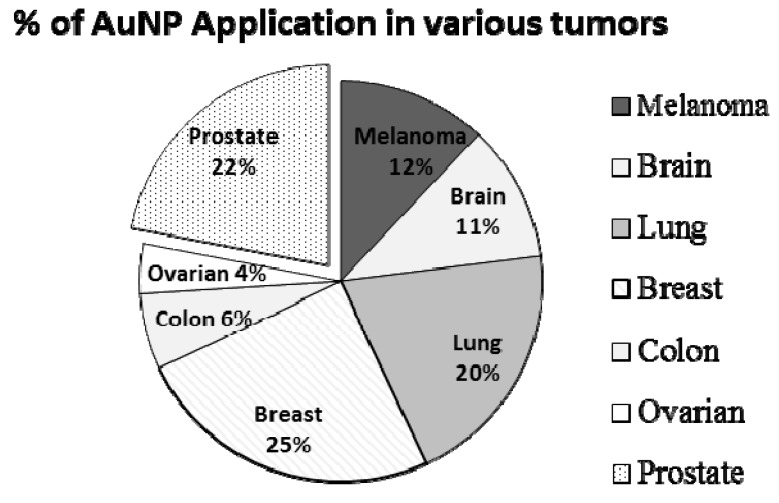
Proportion of gold nanoparticle (AuNP) applications in various types of cancer.

AuNPs can have a large surface bioconjugation property with molecular probes, especially those containing amino, phosphate, and thiol groups [33,34]. Moreover, they also offer particular size and shape-dependent optical properties which are mainly concerned with localized plasmon resonance [35]. In fact, the surface plasmon resonance is a phenomenon that arises when the wavelength of light is much larger than the nanoparticle size and eventually causes the free electrons in the metal to oscillate. As a front wave of light passes, polarization of the electrons on the surface of AuNPs occurs, causing their oscillation in resonance with the light’s frequency [36]. This feature is usually determined by absorption and scattering spectroscopy and is known to depend greatly on the size, shape, and dielectric constants of both the metal and the surrounding material [37]. The highly sensitive surface plasmon resonance (SPR) response is extremely desirable for the reliable measurement of any SPR shift, which signifies changes in the nature of the AuNPs such as efficient molecule binding or an increase in size. Basically, this property allows AuNPs to be used for sensing and bioimaging because SPR shifts are mostly due to changes in the dielectric constants or agglomeration of the particles [38,39]. Therefore, AuNPs can act as strong contrast agents due to their emission spectra which can be 4–5 folds greater than those of efficient fluorophores [40]. Some imaging technique examples enhanced by AuNPs include X-ray, computed tomography, photoacoustic imaging, ultrasound, and many more [41]. Moreover, they can be used as excellent tracking devices for optical imaging and detection of cancerous cells. Lastly, what truly makes AuNPs shine is their ability to be used for photothermal therapy (PTT) of cancers and other disorders such as bacterial infections. Primarily, electron relaxations on the surface of AuNPs are transformed into heat on a time scale of picoseconds [42]. When a light frequency stronger than the SPR absorption of AuNPs is employed (usually infrared light), a hyperthermic environment is generated, and in recent years, the ablation of tumors with hyperthermia from PTT has gained increased interest [43]. All these unique features of AuNPs have been attention grabbers for scientists to grow increasing interest in the research of gold AuNPs in recent years, and this has resulted in having many studies on the applications of AuNPs on different types of cancers, particularly on PCa, breast, lung, brain, and colon cancer, as depicted in Figure 3. Furthermore, a summary of various shapes of AuNPs (such as spheres, rods, cages, etc.) and their applications on PCa is presented in the Table 2.

## 4. Arena of Gold Nanoparticles in PCa

**Figure 4:**
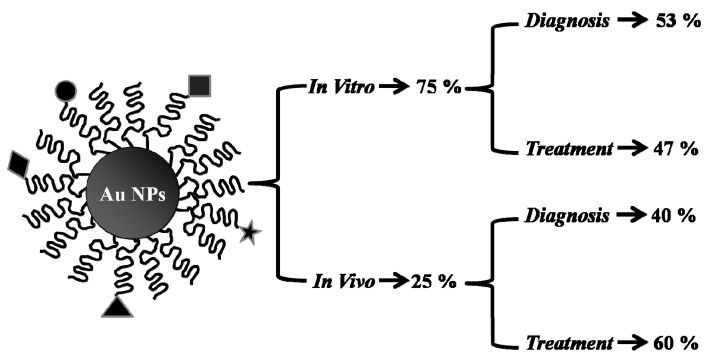
Distribution profile of the studies done using AuNPs on PCa. The majority of the studies done on PCa using AuNPs involved in vitro applications (~75%). Almost half of the studies were dedicated to diagnostic or to treatment purposes, and the majority of the in vivo studies (~60%) involved therapeutic applications. The curved line outside the AuNP represents the stabilizing agent layer that is used to form and stabilize the nanoparticles, and allow for the conjugation of different types of ligands, some of which, for example, could be bifunctional polyethyleneglycol (PEG), polysaccharides, polyethylenimine, or a crosslinker. The circle, lozenge, square, star, and triangle refer to the various types of conjugating ligands (Epigallocatechin gallate, siRNA, antibodies, and folic and anisic acid).

In this review, we describe major studies published regarding different types of AuNPs and their applications in the treatment and diagnosis of prostate cancer. About seven thousand articles have been peer-reviewed and added to the literature database since 1998. A focus on AuNPs that were applied for PCa diagnosis and treatment in the past two decades, from 1998 till 2018, was done. However, we note here that most articles we found were published after the year 2000. The search was restricted to the English language and the type of publication was set to “Journal”, and the output was about 7000 articles. Despite the use of specific keywords, sixty percent of the studies on gold applications involved other types of prostate cancer, such as breast, colorectal, and lung cancers. Another twenty percent contained an adverse gamut of the broad applications of different types of nanoparticles in cancer therapy that were not restricted to gold. The remaining twenty percent were collected as EndNote databases and screened for duplicates. Thus, our aim was to provide an updated review incorporating the latest 20% of articles that match our search criteria and screen them to examine whether all the studies were addressed in the two most recent reviews by Elgvist et al., 2017, “Theranostic Nanoparticle Applications: A Focus on Prostate and Breast Cancer” [9], and by Thambiraj et al., 2018, “An Overview on Applications of Gold Nanoparticle for Early Diagnosis and Targeted Drug Delivery to Prostate Cancer”. Almost 20 articles were not covered by Elgvist’s report and consequently added in this review, and only three articles were overlapping with that of Thambiraj (Table 3). Furthermore, about 75% of all studies were done in vitro, while being split equally between diagnostic and therapeutic approaches (Figure 4). On the other hand, most of the in vivo studies were designed for therapeutic purposes.

### 4.1. Gold Nanoparticles for Diagnosis of PCa

Despite the progress in PCa diagnosis, it is crucial to further develop existing diagnostic mechanisms in order to detect the tumor at its early stages. Many biological markers exist. The prostate specific antigen (PSA), a serum biomarker, is one of the most credible diagnostic and prognostic markers used for PCa detection [44]. The detection limit of PSA is the lowest concentration of PSA that can be detected, and researchers are determinedly pursuing optimal methods that can detect PSA at their lowest levels in order to give a very accurate readout. Doctors often consider PSA levels of 4 ng/mL and lower as normal. Usually, routine PSA blood tests report a minimum of a 4 ng/mL detection limit. This underlines the need of enhanced detection sensitivity through the advantages served by AuNPs. In the following section, we describe various biochemical methods of AuNP modifications used in vitro and in vivo for diagnostic applications that serve a higher sensitivity to detect PSA.

#### 4.1.1. In Vitro Applications

The emergence of nanotechnology, particularly AuNPs, has made improvements soundly achievable due to their unique physiochemical properties and their ability to be used as tags to allow a heightened sensitivity [18].

**Figure 5:**
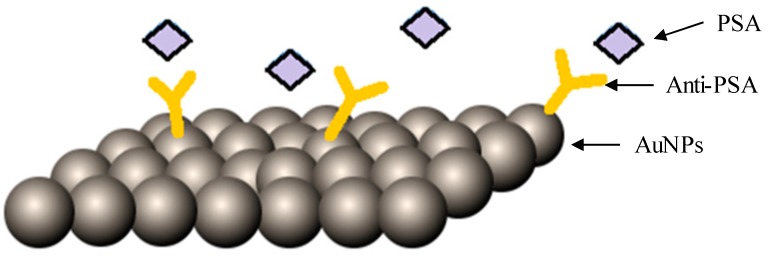
Representative image of 100 nm AuNPs (tethered to an electrode) coated with anti-prostate specific antigen (PSA) antibodies.

In a recent study, Rodriguez et al. (2018) outlined a porous silicon electrode platform tethered to 100 nm gold nanoparticles and coated with anti-PSA antibodies, as depicted in Figure 5. The gold nanoparticles relayed the added value of enhanced conductivity with a remarkable limit of detection, 1 ng/mL PSA [45]. Barbosa et al. (2017) unraveled a new method towards achieving an optimum one-step quantitation of PSA using silver-enhanced gold nanoparticles conjugated to anti-PSA antibodies together with carbon nanoparticles [46]. They aimed at promoting a novel optical detection method using a microcapillary film (MCF) as an immunoassay platform. PSA was successfully quantified in a cost-effective method, presenting a dynamic range of 10 to 100 ng/mL of PSA.

One similar approach that was anticipated for sensitive PSA detection was done by Pal et al. (2017), in which gold nanoparticles supported with graphene oxide (Au–GrO) and attached to anti-PSA antibodies were used as novel biosensors that were applied to human prostate epithelial cells, RWPE-1. The immunosensor resulted in a highly selective and stable model, having a low PSA detection limit of 0.24 fg/mL [47]. Suresh et al. (2018) applied an identical method, however, gold nanoparticles that were coated with a chitosan polymer were used to establish binding to the anti-PSA antibodies [48]. On the other hand, Vural et al. (2018) proposed a method for PSA detection in blood serum samples that involved self-assembled AuNPs with peptide nanotube (PNT) and polyaniline (PANI) composites (PANI/AuNP–PNT) that were used to modify a pencil graphite electrode (PGE). Anti-PSA (Ab1) was immobilized on the modified electrode (PANI/AuNP–PNT/PGE) to capture PSA, and horseradish peroxidase (HRP) labeled anti-PSA (HRP–Ab2) was used as a tracer antibody. The practicality of the novel method was compared to enzyme-linked immunosorbent assay (ELISA) and compatible results were obtained with a minimum limit of detection of 0.68 ng/mL [49]. In a very recent study by Srivastava et al. (2018), the authors also shed light on newly instrumented biosensors composed of graphene quantum dots and goldnanorods (GQDs–AuNRs). Their research involved a comparative study between immunosensors (anti-PSA) and aptasensors (aptamer) bound to the nanoparticles, to efficiently quantitate and detect PSA levels. The performances of both sensors showed comparable results, with an almost same limit of detection (LOD) of 0.14 ng/mL. The aptasensors possessed some advantages over the immunosensors in terms of stability, simplicity, and cost effectiveness [50]. Another model by Huang et al. (2005) showed that using a single domain antigen-binding fragment, such as an anti-PSA VHH camel antibody (cAbPSA–N7) derived from a dromedary heavy-chain antibody, and applying it to a colloidal streptavidin-coated AuNP for PSA detection proved to be a very promising method as well and enabled the detection of PSA levels as low as 1 ng/mL [51].

#### 4.1.2. In Vivo Applications

The inert trait of AuNPs was also exploited as a powerful means for enhanced imaging of PCa in rodents. Harmsen et al. (2017) used 60 nm gold nanoparticles encapsulated with a silica shell and injected intratumorally in mice to achieve a highly sensitive detection with contrast enhanced raman imaging that proves to be promising for future in vivo cancer imaging. The authors also managed to introduce several applications of these nanoprobes for biomedical research, such as intraoperative cancer imaging and a straightforward delineation of cancer without the need for specific biomarker targeting [52]. On the other hand, Sattarahmady et al. (2017) gave insight on a novel method for PSA detection using a round hairbrush-like gold nanostructure conjugated to an aptamer and tested on human blood samples. The fabricated aptamer biosensor was capable of detecting PSA with a minimum limit value of 50 pg/mL [53]. Despite the sensitive range of detection limits achieved, optimizations and refinements in terms of size, coating, and morphology of AuNPs are still needed to achieve an optimum quantification of PSA, since their behavior in vivo mainly depends on these parameters.

### 4.2. Gold Nanoparticles for the Treatment of PCa

Scientists are constantly trying to find effective targeted drug delivery strategies. The real concern they face is to know which method will best act as the “magic bullet” that could relay the desired outcome in terms of gene silencing, cytotoxicity, etc. In many cases, for example, targeting systems such as AuNPs have been designed (through natural extracts that act as active elements in raw substances, antibodies, and ligands such as polypeptides able to recognize a specific receptor that could allow the localization of certain drugs to the diseased environment such as tumors. In the following section, recent models of AuNP modifications applied both in vitro and in vivo are chronologically reported.

#### 4.2.1. In Vitro Applications

Among the AuNPs targeted prostate treatments was a study done by Kastenel et al. (2013), which highlighted the feasibility of efficiently targeting prostate cancer cells with PSMA inhibitor (CTT54)-guided gold nanoparticles. The PSMA-targeted AuNPs exhibited significantly higher and selective binding to LNCaP cells compared to control non-targeted AuNPs in a time-dependent manner [54]. Interestingly, a bigger achievement is met when physics is introduced to biology, giving rise to “photothermal therapy” (PTT). PTT is a minimally invasive technique which uses hyperthermia generated by photothermal agents from laser energy to kill cancer cells [55]. Oh et al. (2015) showed that efficient PCa killing was feasible when photothermal therapy was applied on AuNP clusters by low light irradiation that caused local heating and selective killing of the PC-3 targeted cells [56]. Conversely, Mayle et al. (2016) worked on engineering an A11 minibody conjugated to a gold nanoshell for prostate stem cell antigen (PSCA) in order to facilitate targeted PTT on PSCA-transfected 22Rv1 PCa cells. Results showed significant laser-induced, localized killing of PCa cells in vitro by exhibiting greater efficacy as a PTT agent compared to non-targeted gold nanoshells [57]. Tsai et al. (2016) used AuNPs coated with a tumor-specific green tea natural extract epigallocatechin gallate (EGCg) on metastasized human prostate cancer cells (PC-3), and successfully delivered the chemotherapeutic drug doxorubicin (DOX). Cell proliferation of the PC-3 cells was inhibited concomitantly with enhanced cellular uptake of DOX, as revealed by the calorimetric 3-(4,5-Dimethylthiazol-2-yl)-2,5-diphenyltetrazolium bromide (MTT assay) and based on the selective ability of living cells to reduce MTT into formazan. In addition laser scanning microscopy was used to confirm the MTT results [58]. Butterworth et al. (2016) performed a preclinical evaluation of dithiolated diethylenetriaminepentaacetic acid (DTDTPA)-conjugated AuNPs (Au@DTDTPA) for both CT-contrast enhancement and radiotherapy in PCa [56]. Gold-DTDTPA nanoparticles showed the capability of acting as efficient theranostic agents in prostate cancer by inducing cytotoxicity in the PC-3, DU 145, and PNT2-C2 cells after 24 hrs exposure to the NPs. Moreover, the Au@DTDTPA gave rise to a 10 % CT imaging enhancement. On the other hand, Guo et al. (2016) took advantage of small-interference RNA (siRNA) conjugated to AuNPs to knock down gene expression in prostate cancer cells (PC-3 and LNCaP). Results offered potential applications in transferrin and folate receptor ligands conjugated to AuNPs for prostate cancer treatment [59]. Also, in an article by Fitzgerald et al. (2016), siRNA was successfully conjugated to anisamide-conjugated poly(ethylenimine) (PEI)–AuNPs. Anisamide tends to bind overexpressed sigma receptors in PCa. Results showed that the AuNP complexes resulted in highly efficient knockdown of the *RelA* gene (~70%). The authors also intend to perform folic acid-targeted AuNPs to PCa and other targeting ligands [60]. In fact, the *RelA* gene is a proto-oncogene that encodes the RelA subunit (also known as p65) of the NF-kappa-B (NF-κB) transcription factor, which is involved in many cellular processes and in the progression of many diseases, such as Ependymoma and Reticuloendotheliosis, and most importantly PCa [61]. The activation of NF-κB/RelA has often been correlated with the development of many cancers and have revealed to serve as biomarkers of PCa progression and metastases [62].

A real breakthrough arose when Kim et al. (2017) managed to demonstrate the selective uptake of epidermal growth factor-conjugated gold nanoparticles (EGF–GNP) and how it facilitates non-thermal plasma (NTP)-mediated cell death in prostate DU 145 cells along with other cell lines over-expressing the epidermal growth factor receptor (EGFR). Treatment with the EGF-conjugated GNP complex, followed by NTP irradiation, showed selective apoptosis of cells that have undergone receptor-mediated endocytosis. These results suggest that EGF-conjugated GNP functions as an important adjuvant which gives target specificity in applications of conventional plasma therapy [63].

#### 4.2.2. In Vivo Applications

Similarly, Shukla et al. (2012) injected intratumorally a tumor-specific green tea natural extract, epigallocatechin gallate (EGCg) a most abundant catechin in tea that has a great potential in treating human diseases. EGC functionalized radioactive AuNPs target overexpressed laminin receptors and induce cytototoxic effects, hence circumventing transport barriers, resulting in targeted delivery of therapeutic payloads [31] and resulting in 80% reduction of tumor volumes after 28 days, demonstrating significant inhibition of tumor growth compared to controls. Another promising in vivo study showed up to 80 percent tumor reduction when magniferin radioactive AuNPs, having laminin receptor specificity, were applied in the prostate tumor in severe combined immune deficiency (SCID) mice [64]. Lu et al. (2017) revealed that chrysophanol gold nanoparticles in mice model carry high bioavailability, with sustained releasing properties (30 µg/mL) when introduced intraperitoneally and compared to the free chrysophanol plasma concentration (3 µg/mL) after 2 hrs. Chrysophanol extracts from *Rheum* genus plants have been suggested to alter major signaling pathways leading to cell death in different types of cancer cells [65].

In an interesting study managed by Lechtman et al. (2017), the authors came out with a conclusive finding that there is an interplay between the gold nanoparticle sub-cellular localization (size 1.9 and 100 nm), and the photon energy for radiosensitization in PC-3 prostate cancer cells [66] when incubated with 2 mg/mL of 30 nm AuNPs and irradiated with 100 and 300 kVp beams. Khoo et al. (2017) studied the effect of radiosensitization of prostate cancers in vitro and in vivo to X-rays using actively targeted goserelin-conjugated gold nanorods (gGNRs) [67]. The study showed that treatment of prostate cancer cells with gGNRs promotes gonadotropin-releasing hormone receptor-mediated internalization and enhances radiosensitivity. The in vivo results showed that gGNR treatment, along with x-ray irradiation, is considerably more effective than radiation treatment alone (*p* < 0.0005). This resulted in a striking reduction in tumor volume that was found to be 50% smaller after only 2 months of treatment. Their results provided strong evidence for the feasibility of tumor-specific prostate brachytherapy with gGNRs.

All of these studies highlight the great potential that AuNPs withhold for PCa treatment. However, in order to achieve a bench-to-bedside translation of these great entities, more efforts are still needed to understand their modes of action in biological systems of clinically relevant models, such as monkeys. They will then be truly able to bring us a step closer to demonstrate how these targeted systems function in the human species and whether they invoke other complications that have not been observed in simpler in vivo models.

## 5. Conclusions

Recognizing the growing global prostate cancer crisis, a smart vision is needed to implement nanoparticles as drug carriers. In brief, the significance of AuNPs boils down to the versatile platform they offer through their modifications, which allow them to be conceived as potential contenders in the areas of active tumor targeting and imaging. New avenues will arise through enhanced manipulations of AuNPs. Through modulating the AuNPs’ shape, size, and most importantly, surface characteristics, it becomes possible to fine-tune their properties in order to maximize their applicability as a tool for cancer diagnosis, photothermal therapy, radiotherapy, and targeted gene therapy [68]. It remains quite intriguing to address the effect of different coats on AuNPs on their pharmacokinetics, tissue distribution, and excretion of conjugated AuNPs.

## Figures and Tables

**Table 1:** A comparative table of the various Gold nanoparticle-based treatments for prostate cancer.

Type of NP	Biodistribution and Accumulation	Toxicity	Efficacy of Treatment on PCa	References
Silver	Heart, Lung, Kidney, Liver and Spleen	Size Dependent: dose <10 mg kg^−1^ is safe, while it is toxic when a dose over 20 mg kg^−1^ is adminstered	Moderately to Highly effective (depends on the coating and targeting ligand)	[69,70,71]
Gold	Kidney, Heart, Brain (Size dependant <20nm), Spleen, and Liver (Highest accumulation)	Size, Shape, and Surface coating Dependent for ex: < 50nm and Neutral charged colloidal AuNPs are non-toxic	Highly Effective (Many Modalities exist to induce targeted killing with little or no side effects)	[69,72,73]
Quantum Dots	Kidney, Liver, and Spleen	Low Toxicity (due to incorporation of heavy metals)	Not Applicable (mostly used as biosensors)	[73,74]
Iron Oxide	Liver and Spleen	Low to non-toxic (based on surface charge and coating)	Moderately Effective	[73,75]
Platinum	Liver, Spleen, Kidney, and Lungs	Non- toxic	Not Applicable (only for PSA quantification)	[76,77]
Magnesium Oxide	Liver, Spleen, Stomach, Kidney Brain	Dose-dependent toxicity	Not Applicable	[78,79,80]
Solid lipid	Liver, Heart, Kidney, and Spleen	Non-toxic (must be stabilized by surfactants to form administrable emulsions)	Moderately Effective	[32,81,82]
Carbon	Liver, Spleen, bladder, Intestine	Dose and Route of Administration dependent toxicity	Moderately to Highly Effective (depends on method of treatment)	[83,84,85]
Mesoporous Silica	Liver and Spleen	Non-toxic	Moderately Effective	[86,87,88]
Polymer-based	Liver, Spleen, and Kidney	Low toxicity (based on surface charge)	Highly Effective	[74,89,90]

**Table 2:** Different shapes of gold nanoparticles in prostate cancer.

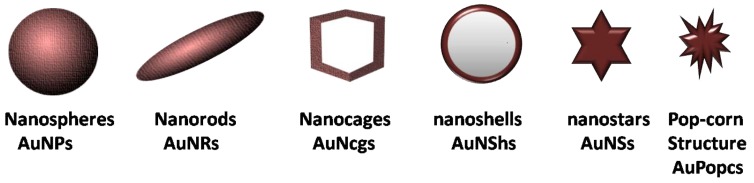 **Various shapes of gold nanostructures used in prostate cancer, some examples are listed below**			
**Gold Nanostructures/Formulation**	**Size**	**Application in Prostate Cancer**	**Reference**
AuNPs-Citrate nanospheres	100 nm	Early stage detection in blood	[91]
AuNPs-Epigallocatechin gallate and gelatin doxorubicin	10–85 nm	DOX release and fluorescence imaging	[58]
AuNPs-Dithiolated diethylenetriamine pentaacetic acid	5.37 nm	Radiotherapy	[92]
AuNPs-PEI-siRNA-Anisamide	8–50 nm	Targeting and gene knockdown	[60]
AuNP-5kPEG-PSMA-1-Pc4	5–25 nm	Targeting and fluorescent photodynamic therapy (PDT)	[93]
AuNRs-Polyethyleneglycol 5KD	60 × 14.8 nm	Plasmonic photothermal therapy (PPTT)	[94]
AuNRs-antibody (Ab-17)	45 × 15 nm	detection by photoacoustic imaging	[95]
AuNRs-Zn(II)-Dipicolylamine-siRNA	84.1 ± 8.6 nm	siRNA Delivery and PPTT	[96]
AuNcgs-Polyethyleneglycol 5KD	50 nm	Plasmonic PPTT	[94]
AuNcgs-Peptides	40-50 nm	Imaging and PPTT	[97]
AuNcgs-CNT hybrid-para-aminothiophenol	—	targeted imaging and PPTT	[98]
AuNShs-Polyethyleneglycol	110 nm silica–10 nm Au shell	Thermal ablation with laser	[99]
AuNShs-PEG-EphrinA1	98–112 nm silica 2–4 nm Au shell	targeted PPTT	[100]
AuNShs	—	Clinical safety profile in human patients	[101]
AuNS-PEG-A10-DUP-1 aptamers	61.9 nm	Ultra-Effective Photothermal Therapy	[102]
AuNS- citrate- polyvinylpyrrolidone	25, 85, 150 nm	Photothermal Therapy	[103]
AuNS-PEG and AuNS@SiO_2_	100 nm	photoacoustic imaging and PPTT	[104]
Nano-popcorn -shaped AuNPs.	4.3–28 nm	diagnosis in LNCaP by surface-enhanced Raman scattering (SERS) an PPTT	[105]

**Table 3:** Summary of the gold nanoparticles employed for the targeted therapy or diagnosis of prostate cancer. The table summarizes the novel strategies employed for prostate cancer theranostics using gold nanoparticles in a chronological order (2005–2018).

Type of AuNPs	Application	Study Type	Diagnosis/Treatment	Target	Result	Reference
anti-PSA camel antibody coated to streptavidin coated AuNPs	A PSA sandwich modified biosensor was used and quantification was done using a surface plasmon resonance instrument.	In vitro	Diagnosis	Not applicable	Major enhancement in sensitivity of PSA detection was observed with a limit of detection as low as 1 ng/mL.	[51]
(EGCg) tagged ^198^AuNPs	PC3-xenograft SCID mice /Intratumorally	In vivo	Treatment	Laminin receptors	80% reduction of tumor volumes after 28 days	[31]
AuNP- biotin-PEG12-CTT54 inhibitor	Prostate cancer cells were targeted with PSMA inhibitor (CTT54)-guided gold NPs.	In vitro	Treatment	PSMA receptor	Higher and selective binding to LNCaP cells compared to control non-targeted AuNPs in a time-dependent manner.	[39]
Phage-AuNP	PC3-cells	In vitro	Treatment	PSMA receptors	Target specific photothermal therapy	[56]
AuNPs-PEG-Tf/AuNPs-PEI-FA.siRNA	LNCap cells /PC3-cells	In vitro	Treatment	Transferrin and Folate receptors	Cellular uptake and non-cytotoxicity of the AuNPs-PEG-Tf was observed. *RelA* gene silencing after 24 h was observed for AuNPs-PEI-FA.siRNA.	[59]
EGCG-AuNPs.DOX	PC3-cells	In vitro	Treatment	Laminin Receptors	Enhanced receptor mediated endocytosis and induction of apoptosis after 24 h	[58]
Au@DTDTPA	CT-contrast imaging and radiotherapy in PC3, DU 145, PNT2-C2 cells, and Human PC3 xenograft tumor models.	In vitro	Treatment and Diagnosis	Not applicable	10 % CT imaging enhancement, increased cytotoxicity after 24 h exposure to the NPs, and tumor growth delay of 17 days.	[92]
A11 minibody-conjugated to a gold nanoshell	Photothermal therapy on PSCA-transfected 22Rv1 prostate cancer cells	In vitro	Treatment	PSCA receptor	Enhanced localized killing of prostate cancer cells compared to nontargeted gold nanoshells.	[57]
GF- ^198^AuNP	CF-1 mice/intratumoral	In vivo	Treatment	Laminin receptors	80% retention of the injected dose (ID) in prostate tumors after 24 h. By three weeks post treatment, over 5 fold reduction of tumor	[64]
Chrysophanol-AuNPs	LNCap/PC3/DU 145	In vitro	Treatment	Not Applicable	Inactivating AKT expression and inducing apoptosis and ROS generation.	[65]
Silver enhanced AuNPs	microfluidic immunoassay precoated with CapAband layered with immobilized gold NPs.	In vitro	Diagnosis	Not applicable	PSA limit of detection range from 10 to 100 ng/mL.	[46]
Au-GrO	Au-GrO on platinum electrode, immobilized with anti PSA	In vitro	Diagnosis	Not applicable	Immunosensor had a PSA limit of detection of 0.24 fg/mL.	[47]
AuNPs encapsulated with a silica shell	Injected intratumorally in Hi-Myc mouse	In vivo	Diagnosis	Not applicable	Highly sensitive tumor detection with contrast-enhanced raman imaging	[52]
Hairbrush-like gold nanostructure	NPs as transducers to fabricate a signal-on built in-marker electrochemical aptasensor for the detection of PSA	In vitro	Diagnosis	Not applicable	The aptasensor detected PSA with a limit of detection of 50 pg mL^−1^.	[53]
EGF-GNP	DU 145 cells	In vitro	Treatment	EGFR receptor	NTP irradiation showed selective apoptosis of cells that have undergone receptor mediated endocytosis.	[63]
gGNRs	Radiotherapy to X-rays using actively targeted gGNRs; applied to mice bearing PC3-xenograft tumors and to PC3 cells	In vitro	Treatment	Not applicable	50% reduction in tumor volume after 2 months of treatment.	[67]
Chitosan-AuNP	A sandwich-type electrochemical immunosensor using anti-PSA was designed for detecting PSA.	In vitro	Diagnosis	Not applicable	The fabricated immunosensor demonstrated excellent sensitivity, stability, and a detection limit of 0.001 ng/mL.	[48]
PANI/AuNP-PNT	Anti-PSA Ab immobilized on modified PANI/AuNP-PNT pencil graphite electrode with HRP-anti PSA antibody to form sandwich immunoassay	In vitro	Diagnosis	Not applicable	Limit of detection was found out to be 0.68 ng/mL	[49]
PSi-GNP	PSA was immobilized at different concentrations on the surface of the sandwich bioassay (NiCr electrode).	In vitro	Diagnosis	Not applicable	Enhanced PSA sensitivity with a limit of detection at 1 ng/mL	[45]
GQDs-AuNRs	Standard PSA solutions were used. NPs immobilized on electrodes tested for efficiency (Anti-PSA.GQDs-AuNRs vs. aptamer-GQDs-AuNRs).	In vitro	Diagnosis	Not applicable	Both had same limit of detection (LOD) of 0.14 ng/mL. The aptasensor advantages over the immunosensor were the stability, simplicity, cost effectiveness.	[50]
